# Ether vs. Chloroform

**Published:** 1870-06

**Authors:** 


Ether vs. Chloroform.—*Mr. Editor:* Two years ago, while listening to some remarks to his class by Prof. W. W. Greene upon Anæsthetics, I made a note of the following, which, in connection with the frequent reports of deaths from chloroform, I should like to see in your journal.

M. A. C.

Portland, Me., December, 1869.

"No man can hesitate a moment with regard to the comparative safety of ether and chloroform. The simple fact is that chloroform frequently kills, and, so far as we know, ether never does. The published reports of deaths from chloroform in the various medical journals average *nearly two a month*, while there is not a well-authenticated case of death from ether on record. With a knowledge of this fact, and also knowing that the majority of these cases have occurred in the hands of skilled and cautious men, the fatal result often taking place during the performance of a trivial operation or before it was begun, I could never feel justified in exposing a patient to the risk of chloroform. During the several years in which I used it I had no trouble, except that in a few instances it so depressed the circulation that I was obliged to withhold it; but no surgeon is justified in quoting his own fortunate exemption from accident as a justification for the use of this agent when in the hands of other men of equal skill, caution and experience, patients, who to the most careful scrutiny present no contra-indication, do every now and then die as if by lightning stroke. With my present convictions upon this subject, if I should lose a patient under chloroform administered upon my own responsibility, I should, in my own conscience, be held guilty of murder. As to mixing the two, I can only say that the more chloroform the more danger; the more ether the greater safety; and I do not believe that any patient can withstand the influence of pure ether administered according to the directions I have given you. In army practice, the imperative necessity for economy, both in time and transportation, probably justifies the selection of the less bulky and more prompt, although dangerous agent, especially in field practice, but I wish to put you on your guard in this connection. It has been asserted over and over again that the abundant statistics of military surgery, both in Europe and America, fail to show any fatal results from chloroform. I am surprised that these statements should pass unchallenged, for in the Peninsular campaign, and during General Grant's operation before Richmond, I saw several instances in which I feel sure that the death of the patient upon or immediately after removal from the operating table was due to chloroform ; and although the attending surgeons referred the result to '*shock*,' superadded to previous exhaustion, my own opinion was corroborated by that of other competent observers ; and I firmly believe, both from these cases and from the somewhat unwilling admissions of certain army surgeons made to me personally, that a thorough and impartial analysis of statistics would show many fatal results from chloroform in military practice.

"The only condition in which chloroform is safe is that of *pain*. As intense suffering neutralizes the effect of opium, so do the pangs of parturition or the terrible twinges of a *tic douloureux* balance the depressing influence of this powerful anæsthetic ; and in such cases its use to the point of relief, and no further, is safe.—*Medical and Surgical Journal.*

